# The Book World of Medicine and Science

**Published:** 1905-08-26

**Authors:** 


### The Book World of Medicine and Science.

Archives of the Middlesex Hospital. Volume V. (Macmillan and Co. Pp. 277.)

The fourth report from the Cancer Research laboratories, which is embodied in this volume, contains much interesting reading. Mr. Lazarus Barlow has written a valuable paper on "Cancer Ages." This is a statistical study based on the notes of patients who have been admitted to the Middlesex Hospital since its earliest records in 1746 down to the end of 1904. As the result of examining the descriptions of 2,073 male and 4,659 female cases of cancer, Mr. Lazarus Barlow has been able to draw the following conclusions:—(1) There is a general tendency for males to be affected by cancer at a later age than females, the mean age of all cancer in males being 55·2, and in females 49·9 years. (2) Cancer of the alimentary tract is about seven times as common in males as in females. (3) The difference in liability of men and of women to cancer of the alimentary tract gradually and uniformly diminishes, as the seat of primary growth is lower in that tract. (4) The mean age of liability of males to cancer of the alimentary tract is 55 years, that of females 52·5 years. (5) In 80 per cent. of all cases of cancer occurring in males the disease affects the alimentary tract. In 80 per cent. of all cases of cancer occurring in females this disease affects the generative tract, including therewith the breast. (6) In both sexes the generative system, whether including or excluding the breast, tends to be the earliest system affected by cancer. In both sexes the system with the latest tendency to be affected by cancer is the cutaneous. In both sexes an intermediate position is held by the alimentary system. (7) No support is afforded by the statistics to the view that the early or late appearance of carcinoma at any given site is determined by the character of the epithelium. (8) Cancer before the age of 35 years is commoner in women than in men, and in both sexes shows a special tendency to affect the generative tract.

In another paper Mr. Campiche and Dr. Lazarus Barlow investigate the pathological and clinical aspects of malignant disease of the breast, taking as their source of information the same accumulated records. There is an interesting note by Dr. Barlow on "Cancer in Iceland." In it he points out that data collected from three regions differing so widely as Iceland, Lahore, and the Middlesex Hospital yield very different information concerning the site incidence of cancer. Whereas cancer of the penis is unknown in Iceland, it is of frequent occurrence amongst the Hindus. Uterine and breast cancer is rare in Iceland as compared with its prevalence in the Middlesex Hospital records. In Iceland cancer most frequently tends to affect the stomach and upper part of the alimentary canal, whereas in India it tends to attack the lower part of the alimentary tract.

The Care and Management of Delicate Children. By Dr. Percy Lewis. (London: Cassell and Company, 1905. Pp. 192. Price 3s. 6d.)

This little book is described as being specially designed for the medical officers of schools, but we think it will also prove useful to a much larger class. Not only will the young practitioner find much that he has never been taught in "the schools," but also the parent of average intelligence will be able to understand and to benefit from the practical teaching. The author recognises how largely children are made delicate, and kept delicate, through the ignorance and folly of parents, and a large part of the book is occupied with the faults in the up-bringing of these children, which must be corrected. There is no doubt that many children attending school are crippled, not through actual disease, but through delicacy, and too often this delicacy has been induced directly through the home life. The various manifestations of this delicacy are passed in review, and the indications for treatment are considered in detail. The therapeutic part of the book, however, does not deal with drugs but with the proper surroundings and habits of life of the child, in reference to his special weakness.

The Maintenance of Health in the Tropics. By W. J. Simpson, M.D., F.R.C.P. (John Bale, Sons and Danielsson. 1905. Pp. 118, Illustrations 10. Price 2s. 6d. net.)

This little manual has been published, under the auspices of the London School of Tropical Medicine, specially for the use and guidance of the general public living in hot climates. The directions are simple and clear, and as no technical terms are used the book is intelligible to all. Personal hygiene is discussed at some length, and advice concerning climate, diet, drinking-water and dwelling houses is fully given. The causation and prevention of malaria are clearly described, the life history of the mosquito being explained with the aid of several helpful diagrams. The symptoms of and the preventive measures against the other more important tropical diseases are fully dealt with.

The Health of the Nation. A Letter to the Local Government Board. By J. Theodore Dodd, B.A. (Oxford: Bocardo Press. 1905. Pp. 36. Price 6d. net.)

The author is evidently a hard-working and enthusiastic Guardian, who has carefully studied poor-law problems. In his letter, which is moderate in its tone, he advocates co-operation of the Guardians with Town Councils, the utilisation to their full extent of all powers granted by the Local Government Board to Guardians, and urges that nothing be put in the way of the sick poor when seeking outdoor medical relief. Several other points are discussed, and for all those who are interested in poor-law the letter is well worth reading.

Dispensing Made Easy. By W. G. Sutherland, M.B. Aberd. Second Edition. (Bristol: John Wright and Co. 1905. Pp. 104. Price 3s. 6d. net.)

We are glad to have the opportunity of commending a second edition of this work to the medical profession. It is a most admirable guide to economical and efficient dispensing, and with its help most dispensing medical practitioners would be able to save themselves a good deal of labour and, very likely, several pounds a year in the drug bill.

The Matriculation Directory. June 1905. (W. B. Clive. Pp. 146. 1s. net.)

This consists of a complete set of the papers set in the London Matriculation of June 1905, with their solutions. These solutions, intended to serve as models to prospective candidates, are clear and reliable. Diagrams are given where necessary. A helpful criticism of the papers closes the volume, which we commend to all interested in the London Matriculation.

